# MOG-IgG in NMO and related disorders: a multicenter study of 50 patients. Part 1: Frequency, syndrome specificity, influence of disease activity, long-term course, association with AQP4-IgG, and origin

**DOI:** 10.1186/s12974-016-0717-1

**Published:** 2016-09-26

**Authors:** Sven Jarius, Klemens Ruprecht, Ingo Kleiter, Nadja Borisow, Nasrin Asgari, Kalliopi Pitarokoili, Florence Pache, Oliver Stich, Lena-Alexandra Beume, Martin W. Hümmert, Corinna Trebst, Marius Ringelstein, Orhan Aktas, Alexander Winkelmann, Mathias Buttmann, Alexander Schwarz, Hanna Zimmermann, Alexander U. Brandt, Diego Franciotta, Marco Capobianco, Joseph Kuchling, Jürgen Haas, Mirjam Korporal-Kuhnke, Soeren Thue Lillevang, Kai Fechner, Kathrin Schanda, Friedemann Paul, Brigitte Wildemann, Markus Reindl, in cooperation with the Neuromyelitis Optica Study Group (NEMOS)

**Affiliations:** 1 Molecular Neuroimmunology Group, Otto Meyerhof Center, Department of Neurology, University Hospital Heidelberg, Im Neuenheimer Feld 350, 69120 Heidelberg, Germany; 2 Department of Neurology, Charité-University Medicine Berlin, Berlin, Germany; 3 Department of Neurology, Ruhr University Bochum, Bochum, Germany; 4 NeuroCure Clinical Research Center and Clinical and Experimental Multiple Sclerosis Research Center, Department of Neurology, Charité University Medicine, Berlin, Germany; 5 Experimental and Clinical Research Center, Max Delbrueck Center for Molecular Medicine, Charité University Medicine Berlin, Berlin, Germany; 6 Department of Neurology and Institute of Molecular Medicine, University of Southern Denmark, Odense, Denmark; 7 Department of Neurology, Albert Ludwigs University, Freiburg, Germany; 8 Department of Neurology, Hannover Medical School, Hannover, Germany; 9 Department of Neurology, Heinrich Heine University, Düsseldorf, Germany; 10 Department of Neurology, University of Rostock, Rostock, Germany; 11 Department of Neurology, Julius Maximilians University, Würzburg, Germany; 12 IRCCS, C. Mondino National Neurological Institute, Pavia, Italy; 13 Centro di Riferimento Regionale SM, Azienda Ospedaliero Universitaria San Luigi Gonzaga, Orbassano, Italy; 14 Department of Clinical Immunology, Odense University Hospital, Odense, Denmark; 15 Institute of Experimental Immunology, affiliated to Euroimmun AG, Lübeck, Germany; 16 Clinical Department of Neurology, Medical University Innsbruck, Innsbruck, Austria

**Keywords:** Neuromyelitis optica (NMO), Devic’s syndrome, Optic neuritis, Transverse Myelitis, Longitudinally extensive transverse myelitis (LETM), Neuromyelitis optica spectrum disorders (NMOSD), Multiple sclerosis, Autoantibodies, Myelin oligodendrocyte glycoprotein antibodies (MOG-IgG), Neuromyelitis optica antibodies (NMO-IgG), Aquaporin-4 antibodies (AQP4-IgG), Cell-based assays, Cerebrospinal fluid, Antibody index

## Abstract

**Background:**

Antibodies to myelin oligodendrocyte glycoprotein (MOG-IgG) have been suggested to play a role in a subset of patients with neuromyelitis optica and related disorders.

**Objective:**

To assess (i) the frequency of MOG-IgG in a large and predominantly Caucasian cohort of patients with optic neuritis (ON) and/or myelitis; (ii) the frequency of MOG-IgG among AQP4-IgG-positive patients and vice versa; (iii) the origin and frequency of MOG-IgG in the cerebrospinal fluid (CSF); (iv) the presence of MOG-IgG at disease onset; and (v) the influence of disease activity and treatment status on MOG-IgG titers.

**Methods:**

614 serum samples from patients with ON and/or myelitis and from controls, including 92 follow-up samples from 55 subjects, and 18 CSF samples were tested for MOG-IgG using a live cell-based assay (CBA) employing full-length human MOG-transfected HEK293A cells.

**Results:**

MOG-IgG was detected in 95 sera from 50 patients with ON and/or myelitis, including 22/54 (40.7 %) patients with a history of both ON and myelitis, 22/103 (21.4 %) with a history of ON but no myelitis and 6/45 (13.3 %) with a history of longitudinally extensive transverse myelitis but no ON, and in 1 control patient with encephalitis and a connective tissue disorder, all of whom were negative for AQP4-IgG. MOG-IgG was absent in 221 further controls, including 83 patients with AQP4-IgG-seropositive neuromyelitis optica spectrum disorders and 85 with multiple sclerosis (MS). MOG-IgG was found in 12/18 (67 %) CSF samples from MOG-IgG-seropositive patients; the MOG-IgG-specific antibody index was negative in all cases, indicating a predominantly peripheral origin of CSF MOG-IgG. Serum and CSF MOG-IgG belonged to the complement-activating IgG1 subclass. MOG-IgG was present already at disease onset. The antibodies remained detectable in 40/45 (89 %) follow-up samples obtained over a median period of 16.5 months (range 0–123). Serum titers were higher during attacks than during remission (*p* < 0.0001), highest during attacks of simultaneous myelitis and ON, lowest during acute isolated ON, and declined following treatment.

**Conclusions:**

To date, this is the largest cohort studied for IgG to human full-length MOG by means of an up-to-date CBA. MOG-IgG is present in a substantial subset of patients with ON and/or myelitis, but not in classical MS. Co-existence of MOG-IgG and AQP4-IgG is highly uncommon. CSF MOG-IgG is of extrathecal origin. Serum MOG-IgG is present already at disease onset and remains detectable in the long-term course. Serum titers depend on disease activity and treatment status.

## Background

Neuromyelitis optica (NMO) is a severely disabling autoimmune disorder of the CNS. In the majority of cases, NMO is caused by autoantibodies to aquaporin-4 (AQP4-IgG) [1–6]; however, 10–20 % of patients with NMO are negative for AQP4-IgG [7–11]. Back in 2007, based on preliminary results, we and others suggested a potential role for IgG antibodies to myelin oligodendrocyte glycoprotein (MOG-IgG) in AQP4-IgG-seronegative NMO. At that time, however, MOG-IgG were still detected by enzyme-linked immunosorbent assays (ELISA) or immunoprecipitation assays, methods that were not always reliable [12], and skepticism prevailed. The following years saw the rise of so-called cell-based assays (CBA) for the detection of autoantibodies. CBA have shown excellent sensitivity and specificity in many applications, including AQP4-IgG testing [8, 9]. Briefly, cultured human cells (mostly HEK293 cells) are transfected with the antigen of interest not constitutively expressed in those cells and used as antigenic substrate in an indirect immunofluorescence assay; mock-transfected cells are used as internal controls. According to a recent consensus statement, CBA are currently considered the best method for detecting AQP4-IgG in NMO [9, 13]. Moreover, assays for detecting conformation-sensitive antibodies to MOG were devised. By the use of such assays, several groups have demonstrated antibodies to MOG in mostly pediatric patients with ADEM or MS-like disease [14–16].

Later on, in a study published in this journal in 2011, some of us demonstrated antibodies to full-length MOG in patients with NMO for the first time by means of a CBA [17]. In the meantime, several studies by us and others have confirmed the association of MOG-IgG with NMO and with related disorders such as isolated optic neuritis (ON) or myelitis [18–28]. Most studies have found MOG-IgG exclusively in patients with ON and/or myelitis who are negative for AQP4-IgG, suggesting that MOG-IgG may denote a disease entity in its own right. The latter notion is further supported by recent in vitro and in vivo studies suggesting a direct pathogenic role of MOG-IgG [17, 29] and by studies demonstrating substantial differences in the histopathology of AQP4-IgG- and MOG-IgG-associated CNS lesions [30–33].

However, there were some obvious limitations: First, many of the previously investigated cohorts were relatively small. Second, long-term data were often absent, with follow-up samples not being available. Third, some cohorts included no Caucasian patients or were genetically mixed, which may be of relevance since genetic factors are thought to play a role in NMO [34]. Fourth, some cohorts were preselected according to AQP4-IgG serostatus. Fifth, control groups in some previous studies were formally too small to assess the specificity of antibody results in a reliable way. Sixth, these last two limitations prompt uncertainty about the prevalence of the rare so-called ‘double-positive’ samples, i.e., samples positive for both NMO-IgG and AQP4-IgG, that have been reported in a few studies [17, 22, 35]. Finally, most previous investigations have focused on serum and included no or only few cerebrospinal fluid (CSF) samples.

In the present study we assessed the frequency of MOG-IgG as assessed by means of a live-cell CBA [17] (i) in a large series of samples from predominantly Caucasian patients sent in for AQP4-IgG and MOG-IgG testing and (ii) in a well-defined cohort of Caucasian control patients with multiple sclerosis (MS) and other inflammatory CNS disorders as well as in healthy controls (*N* = 614). In addition, we evaluated (iii) the prevalence of MOG-IgG and AQP4-IgG double positivity based on a very large number of samples (*N* = 459); (iv) the presence of MOG-IgG at disease onset; (v) the long-term persistence of MOG-IgG in individual patients; (vi) the influence of disease activity and treatment status on MOG-IgG titers; and (vii) the frequency and origin of MOG-IgG antibodies in the CSF.

This study is part of an article series on MOG-IgG in CNS inflammation. In part 2, we systematically evaluate the clinical and paraclinical features present in MOG-IgG-positive ON and/or myelitis as well as treatment responses and long-term outcome [36]. In part 3, we analyze the clinical and radiological features, course, and prognosis of patients with MOG-IgG-associated brainstem encephalitis [37]. In part 4, we report on the frequency and severity of afferent visual nerve damage in MOG-IgG-associated ON as detected by retinal optical coherence tomography (OCT) [38].

## Methods

In total, 614 serum samples and 18 CSF samples from 522 subjects were tested for MOG-IgG. Group I comprised 386 serum samples from 300 patients referred for routine MOG-IgG testing by 11 European academic centers, including the departments of neurology at the University of Heidelberg, the Charité-University Medicine Berlin, the University of Düsseldorf, the University of Bochum, Hannover Medical School, the University of Würzburg, the University of Rostock, the University of Freiburg, all in Germany; the University of Southern Denmark, Denmark; the MS Center at the Azienda Ospedaliero Universitaria San Luigi Gonzaga, Orbassano, Italy; and the IRCCS, C. Mondino National Neurological Institute, Pavia, Italy; eight of which are members of the German Neuromyelitis optica Study Group (NEMOS). Samples were taken for routine clinical assessment. Diagnoses at the time of blood sampling as reported by the referring centers, all of which were tertiary care university hospitals with specialized neuroimmunological departments, included “ON and myelitis” in 54 patients (1 x AQP4-IgG-positive;﻿ 79 serum samples available for testing), “monophasic ON” in 66 (69 samples), “recurrent ON” in 37 (median number of ON attacks 4, range 2–15; 76 samples), “longitudinally extensive transverse myelitis” in 45 (57 samples), “relapsing remitting MS” (RRMS) in 50 (54 samples), “secondary progressive MS” (SPMS) in 2 (2 samples), “primary progressive MS” (PPMS) in 2 (2 samples), and “other neurological disorder” (OND) in 44 (47 samples).

Group II consisted of 89 anonymized serum samples from 83 control patients with AQP4-IgG-positive ON and/or myelitis. Of those, 56 had a history of ON and myelitis, 22 of myelitis but no ON, and 5 of ON but no myelitis. AQP4-IgG had been previously detected by use of a commercial CBA (Euroimmun, Lübeck, Germany) in these patients [8] and by means of an ELISA (RSR, Cardiff, UK) [10].

Group III was made up of 85 anonymized serum samples from 85 control patients with MS according to the McDonald criteria (RRMS in 73, SPMS in 9, PPMS in 3).

Group IV comprised 54 anonymized samples from 9 control patients with OND (including 8 with connective tissue disorders and brain involvement [39]) and from 45 healthy controls.

Ninety-two follow-up samples (86 × group I, 6 × controls) from 55 subjects were tested. The sex ratios (m:f) were 1:2.4 in group I and 1:3 in the control groups II–IV. The median age was 39 years in group I and 38 years among the control patients (groups II–IV). See Table 1 for additional demographic data. 516/522 (98.9 %) tested subjects were of Caucasian descent, including 298/300 (99.3 %) in group I.

**Table 1:** Demographic and serological findings from 522 subjects and 614 serum samples tested for MOG-IgG

Diagnostic categories	Sample numbers	Patient numbers	Sex ratio (m:f)	Age (ys), median	MOG-IgG+, samples	MOG-IgG+, patients	MOG-IgG+, median^§^	AQP4-IgG +, MOG-IgG + patients
Group I	386	300	1:2.4	39	95/386 (24.6 %)	50/300 (16.7 %)	1:640	0/50 (0 %)
“ON and/or MY”^a^	281	202			95/281 (33.8 %)	50/202 (24.8 %)	1:640	0/50 (0 %)
“ON and MY”^a^	79	54			39/79 (49.4 %)	22/54 (40.7 %)	1:1280	0/22 (0 %)
“mON/rON”^a^	145	103			47/145 (32.4 %)	22/103 (21.4 %)	1:640	0/22 (0 %)
“mON”^a^	69	66			10/69 (14.5 %)	9/66 (13.6 %)	1:800	0/9 (0 %)
“rON”^a^	76	37			37/76 (48.7 %)	13/37 (35.1 %)	1:640	0/13 (0 %)
“MY” (all LETM)^a^	57	45			9/57 (15.8 %)	6/45 (13.3 %)	1:2560	0/6 (0 %)
“MS”^a^	58	54			0/58 (0 %)	0/54 (0 %)	N.a.	N.a.
“OND”^a^	47	44			0/47 (0 %)	0/44 (0 %)	N.a.	N.a.
Group II	89	83	1:1.9	46	0/89 (0 %)	0/83 (0 %)	N.a.	89/89 (100 %)
AQP4+ NMO	59	56			0/59 (0 %)	0/56 (0 %)	N.a.	59/59 (100 %)
AQP4+ rON	5	5			0/25 (0 %)	0/22 (0 %)	N.a.	25/25 (100 %)
AQP4+ LETM	25	22			0/5 (0 %)	0/5 (0 %)	N.a.	5/5 (100 %)
Group III	85	85	1:3	38	0/85 (0 %)	0/85 (0 %)	N.a.	N.a.
RRMS	73	73			0/73 (0 %)	0/73 (0 %)	N.a.	N.a.
SPMS	9	9			0/9 (0 %)	0/9 (0 %)	N.a.	N.a.
PPMS	3	3			0/3 (0 %)	0/3 (0 %)	N.a.	N.a.
Group IV	54	54	1:1.3	38	1/54 (1.9 %)	1/54 (1.9 %)	1:320*	0/1 (0 %)
OND	9	9			1/9 (11.1 %)	1/9 (11.1 %)	1:320*	0/1 (0 %)
HC	45	45			0/45 (0 %)	0/45 (0 %)	N.a.	N.a.
Group II–IV	228	222	1:1.9	38	1/228 (0.5 %)	1/222 (0.5 %)	1:320*	0/1 (0 %)
Total	614	522	1:2.6	38	96/614 (15.6 %)	51/522 (9.8 %)	1:640	0/51 (0 %)

*N.a* not applicable, *ON* optic neuritis, *mON* monophasic ON, *rON* recurrent ON, *MY* myelitis, *LETM* longitudinally extensive transverse myelitis, *MS* multiple sclerosis, *OND* other neurological disorders, *RRMS* relapsing remitting MS, *SPMS* secondary progressive MS, *PPMS* primary progressive MS, *HC* healthy control. ^a^Suspected diagnosis at the time of sample referral. ^§^MOG-IgG-positive samples only. *Single patient

All sera were tested using a live-cell CBA employing HEK293A cells transfected with full-length human MOG as previously described [17]. Screening of serum samples was performed at dilutions of 1:20 and 1:40, and antibody titers of positive serum samples were determined by serial dilutions. MOG-antibody titers of ≥1:160 were classified as seropositive [17]. If samples were tested more than once, the highest titer obtained with each sample was used for analysis in all control groups to ensure that data on assay specificity were as conservative as possible. Low-titer results (1:160–1:320) were confirmed in a second, methodologically independent CBA employing formalin-fixed HEK293 cells transfected with full-length human MOG (Euroimmun). CSF samples were screened undiluted, and antibody titers of positive samples were determined by serial dilutions (1:2, 1:4, etc.). The control samples were tested with MOG-IgG-positive serum samples interspersed. MOG-IgG serostatus and titers were determined by two independent investigators blinded to all clinical data (M.R., K.S.). To assess the origin of CSF MOG-IgG, the MOG-specific antibody index (AI_MOG_) was determined. Calculation of AIs allows quantification of antigen-specific intrathecal antibody synthesis [40–43]. Briefly, AI_MOG_ values were calculated as the ratio between the CSF/serum quotient for MOG-IgG, Q_MOG-IgG_, and the CSF/serum quotient for total IgG, Q_IgG(total)_, or Q_lim_, if Q_IgG(total)_ exceeded Q_lim_; i.e., AI_MOG_ = Q_MOG-IgG_/Q_IgG(total)_, if Q_IgG(total)_ < Q_lim_, and AI_MOG_ = Q_MOG-IgG_/Q_lim_, if Q_IgG(total)_ > Q_lim_. CSF and serum samples were obtained at the same time. Usually, values >1.5 are considered as evidence of intrathecal specific antibody synthesis [40, 41]. However, if titers instead of concentrations are used to calculate the AI, a cut-off value of 4 has been recommended [44]. Reiber’s empiric hyperbolic function Q_lim_ was applied to control for possible underestimation of intrathecal specific synthesis due to disturbances of the blood-CSF barrier function and was calculated as follows [45]: ${\mathsf{Q}}_{\mathsf{lim}\left(\mathsf{I}\mathsf{g}\mathsf{G}\right)}=\mathsf{0}.\mathsf{93}\sqrt{{\left({\mathsf{Q}}_{\mathsf{Alb}}\right)}^{\mathsf{2}}+\mathsf{6}\times \mathsf{1}{\mathsf{0}}^{{\textstyle \hbox{-}}\mathsf{6}}}-\mathsf{1}.\mathsf{7}\times \mathsf{1}{\mathsf{0}}^{{\textstyle \hbox{-}}\mathsf{3}}$

The study was approved by the institutional review boards of the participating centers and patients gave their informed consent for publication of clinical data. The control samples were tested in anonymized fashion as requested by the institutional review board of the University of Heidelberg. The Mann-Whitney *U* test was used to compare antibody titers between groups, and the Kruskal-Wallis test with Dunn’s post test to compare more than two groups. Differences with *P* values <0.05 were considered statistically significant.

## Results

### Frequency of serum MOG-IgG and syndrome specificity

**Fig. 1:**
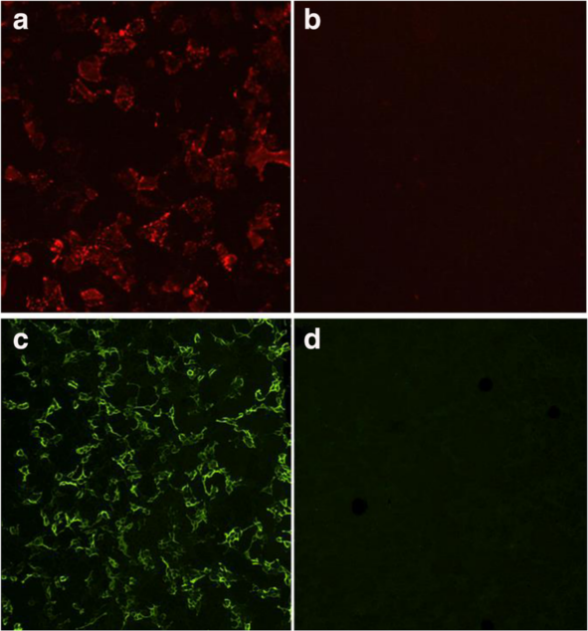
MOG-IgG as detected by two independent cell-based assays (CBA): typical findings. **a**, **b** Binding of serum IgG from a group I patient (**a**) but not from a control patient (**b**) to live HEK293A cells transfected with human full-length MOG. **c**, **d** Binding of serum IgG from a group I patient to formalin-fixed HEK293 cells transfected with full-length MOG (**c**) but not to their mock-transfected counterpart (**d**) in a commercial CBA. Bound MOG-IgG was visualized in the live-cell assay using a Cy3-conjugated goat anti-human IgG antibody and in the fixed-cell assay by use of a fluorescein isothiocyanate (FITC)-labeled goat anti-human IgG antibody

**Fig. 2:**
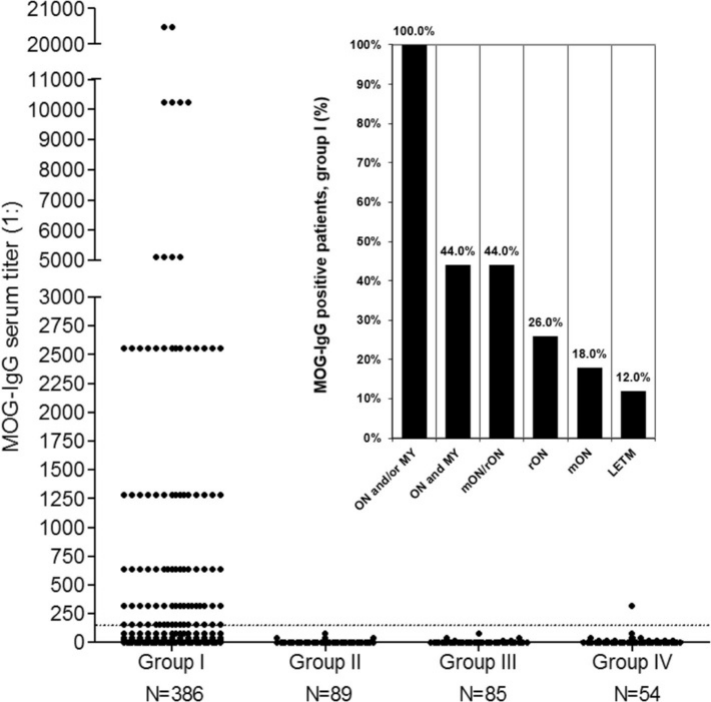
Frequency and titers of MOG-IgG in 614 serum samples from 522 subjects as detected using a live-cell CBA. MOG-IgG was detected in 95/386 (24.6 %) samples in group I but was almost completely absent among 228 control samples (groups II–IV), including 89 samples from AQP4-IgG-positive patients, 85 samples from patients with MS according to the McDonald criteria (group III), and 54 samples from healthy controls and OND patients (group IV). While all low-titer samples (1:160–1:320) in group I were positive also in the fixed-cell CBA, the only positive control sample (from group IV) was negative in the fixed-cell CBA, suggesting a false-positive test result. The *horizontal dashed line* indicates the assay-specific cut-off (> = 1:160)

Overall, 96/614 (15.6 %) samples and 51/522 (9.8 %) subjects were positive for MOG-IgG (Figs. 1 and 2). In group I (samples sent in for routine assessment of MOG-IgG), MOG-IgG was detected in 95/386 (24.6 %) samples from 50/300 (16.7 %) patients; if only patients with a diagnosis of ON and/or myelitis are considered, MOG-IgG was present in 95/281 (33.8 %) samples from 50/202 (24.8 %) patients. In group II (AQP4-IgG-positive controls), none of 89 samples from 83 patients was positive for MOG-IgG. MOG-IgG was also absent in 85 samples from 85 patients in group III (MS control samples). In group IV (OND and healthy controls), 1/54 (1.9 %) samples from 1/54 (1.9 %) patients was positive for MOG-IgG (Fig. 2). In total, MOG-IgG was present in 1 of 228 (0.4 %) control samples or 1 of 222 (0.5 %) control patients (*p* < 0.0001 for group I patients vs groups II–IV patients).

**Fig. 3:**
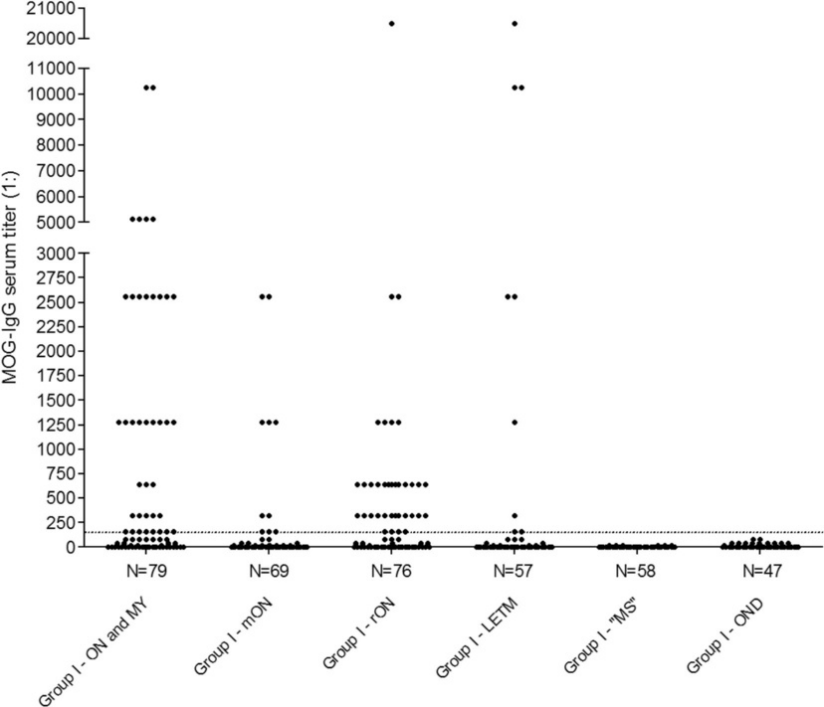
MOG-IgG serum titers in 386 samples from 300 patients included in group I. Diagnoses are given as provided by the referring centers. ON and MY = optic neuritis and myelitis; mON = monophasic optic neuritis; rON = recurrent ON; LETM = longitudinally extensive transverse myelitis; MS = multiple sclerosis; OND = other neurological disorders

All MOG-IgG-positive patients in group I had a history of ON and/or myelitis (Table 1, Fig. 3); 22/50 (44 %) had a history of both ON and myelitis; 22/50 (44 %) had a history of ON but not of myelitis (recurrent in 13); and 6/50 (12 %) had a history of longitudinally extensive myelitis (LETM) but not of ON. The relative frequencies of MOG-IgG in group I patients with a history of ON and myelitis, myelitis but not ON, and ON but not myelitis, respectively, were 22/54 (40.7 %), 22/103 (21.4 %), and 6/45 (13.3 %). All of the MOG-IgG-positive patients were negative for AQP4-IgG. Detailed clinical, radiological, electrophysiological, and laboratory data as well as data on treatment responses and outcome are reported in parts 2, 3 and 4 of this series [36–38]. Moreover, detailed case reports can be found in the *Appendix* sections of part 2 [36] and part 3 [37].

The only positive control sample was a low-titer sample (1 × 1:320, re-testing: 1 × 1:160) obtained from an OND patient from group IV originally diagnosed with systemic lupus erythematosus (American College of Rheumatology criteria met) and “leukoencephalitis of unknown origin”. Symptoms included “scotoma”, “seizures” and “depression”; the sample was negative when tested in the fixed-cell CBA used to confirm the other low titer samples, suggesting a possible false-positive result. As the control samples were analyzed in anonymized fashion, no more data were available on this case. By contrast, 11 further samples from 11 patients with CNS symptoms and systemic lupus erythematosus or other connective tissue disorders included in groups I and IV were negative for MOG-IgG. Follow-up samples were available from 6 MOG-IgG-negative control patients (groups II–IV), all of which were also negative for MOG-IgG.

### Co-existence of MOG-IgG and AQP4-IgG

None of the 51 MOG-IgG-positive group I and IV patients was positive for AQP4-IgG, and none of 84 AQP4-IgG-positive patients from groups II and I was positive for MOG-IgG (Table 1). AQP4-IgG was tested in MOG-IgG-positive patients using a standardized commercial CBA [8] in 48 (94 %) and by ELISA [10] in 3 (6 %). In addition, 226 patients from group I and 98 control patients from groups III and IV were negative both for MOG-IgG and for AQP4-IgG. Overall, 459 patients were tested for both MOG-IgG and AQP4-IgG.

### MOG-IgG serum titers

If all seropositive samples are considered, MOG-IgG titers in group I as determined in the live CBA ranged between 1:160 and 1:20480. If only the highest titer sample in each patient is considered, the median titer in group I was 1:1280 (range 160–20480; *N* = 50); maximum titres were higher in patients with a history of myelitis (median 1:2560, range 160-20480; *N*=7) or a history of both myelitis and ON (1:1280; range 160-10240; *N*=22) at last follow-up than in patients with a history of ON but no myelitis at last follow-up (1:640; range 160-20480; *N*=21), but the difference did not reach statistical significance.

### Presence of serum MOG-IgG at disease onset

MOG-IgG was present already at disease onset in all patients with available data: 2 MOG-IgG positive sera were taken within the first week (at 2 and 4 days) after disease onset, 10 within the first month (median 10 days after onset, range 2–31), and 18 within the first 3 months (median 26 days after onset, range 2–85). The median MOG-IgG titer at disease onset was 1:2560 (range 160–20480; *N* = 18).

### Persistence of serum MOG-IgG in the long-term course

In 18/22 (81.8 %) patients with follow-up samples, all available samples were positive; in the remaining 4 patients, MOG-IgG turned negative at least once. Overall, 40 (89 %) of 45 follow-up samples from MOG-IgG-positive patients with ON and/or myelitis were positive after a median interval between first and last sampling of 16.5 months (range 0–123). 13/13 (100 %) patients were still positive for MOG-IgG 1 year after the initial sample was taken, 8/8 (100 %) 2 years after the initial sample, and 5/5 (100 %) after 4 years. From three patients, stored samples obtained 6.5 years, 8.5 years, and more than 10 years before the last sample (and 13, 11, and 8.5 years after disease onset) were available for retrospective testing and were positive as well (first sample 1:1280 and last sample 1:640 in two patients; 1:320 and 1:160 in the third).

9/11 (81.8 %) patients that were positive for MOG-IgG during an acute attack and had at least one available follow-up sample obtained during remission remained positive during remission. In one of these patients, MOG-IgG titers temporarily fell below the cut-off once during remission (1:80; cut-off 1:160); however, five additional follow-up samples from the same patient obtained during remission were all positive. Similarly, titers were below the cut-off in two follow-up samples (2 × 1:80) taken during remission in another patient but were again positive (1:320) at last follow-up.

Follow-up samples obtained after plasma exchange (PEX) or immunoadsorption (IA) were available from two patients. In the first patient, titers declined from 1:10240 to 1:640 and subsequently disappeared completely after treatment with intravenous methylprednisolone (IVMP), PEX, intravenous immunoglobulins (IVIG), and oral steroids. In the second case, titers declined from 1:5120 to 1:20 after 5 cycles of IA.

Thirty-four follow-up samples were obtained from patients with ON and/or myelitis from group I who were negative at first testing; all of them were negative for MOG-IgG as well.

### Impact of disease activity on MOG-IgG serum titers

**Fig. 4:**
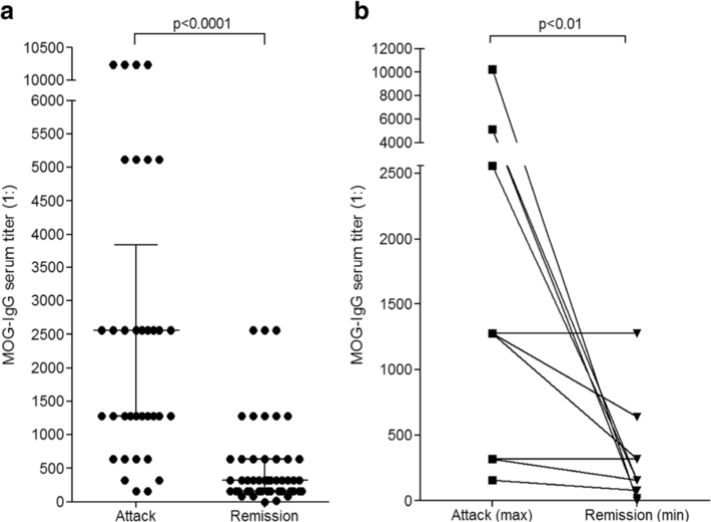
MOG-IgG titers and disease activity. Titers were significantly higher during acute attacks than during remission in the total cohort (**a**) as well as in individual patients with available follow-up sera (**b**). Horizontal lines and whiskers in panel **a** indicate median titers and interquartile ranges, respectively. The median interval between samples in the right panel was 16.5 months (range 2–103). Note that panel **b** shows maximum titers detected during acute attacks and minimum titers detected in follow-up sera. The difference was also significant if not the remission sample with the lowest titer but that with the longest time interval since attack onset was used (median 1:1280 vs. 1:320; *p* < 0.009; not shown)

36/85 (42.4 %) MOG-IgG positive samples with available data were taken within 60 days after an acute attack. Median MOG-IgG titers were significantly higher (1:2560) in samples taken at the time of onset of an acute attack or shortly thereafter (median 14 days; *N* = 33) than in those taken during remission (>60 days since attack onset; 1:320; *N* = 44) (*p* < 0.0001) (Fig. 4a). MOG-IgG titers also differed significantly between acute attacks and remission in individual patients (Fig. 4b). However, titers observed during acute attacks varied both intra- and interindividually (interquartile range 1:1280–3200; absolute range 160–20480), and relatively high titers were also found in a few samples obtained during remission (interquartile range 1:160–640; absolute range 0–2560).

### Impact of clinical presentation on MOG-IgG serum titers during acute attacks

**Fig. 5:**
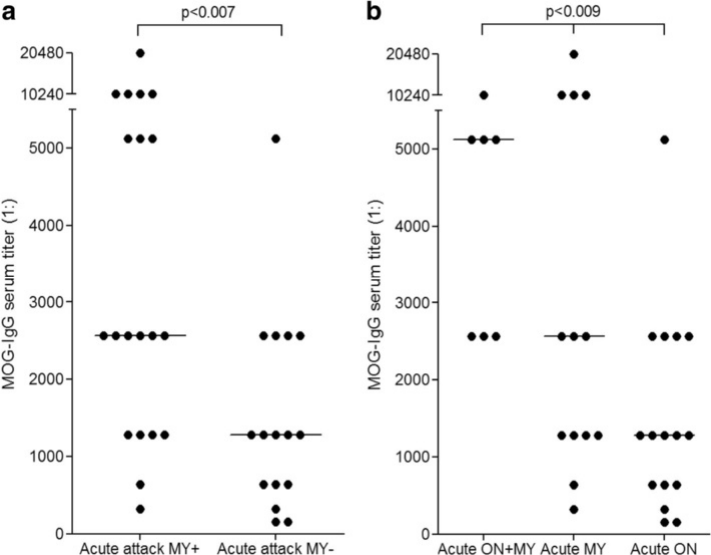
MOG-IgG titers and clinical presentation. Titers were higher during attacks involving myelitis than in attacks not involving myelitis (**a**), and higher during attacks involving simultaneous ON and myelitis than in attacks of isolated myelitis or isolated ON (**b**). The horizontal lines indicate median titers. ON = optic neuritis; MY = myelitis

Median MOG-IgG titers were slightly higher during attacks involving acute myelitis (1:2560, range 320–20480; *N* = 20) than during attacks not involving acute myelitis (1:1280, range 160–5120; *N* = 16; *p* < 0.007) (Fig. 5a). Moreover, median titers were higher during attacks involving simultaneous myelitis and ON (1:5120, range 2560–10240; *N* = 7; additional brainstem encephalitis in three) than during attacks involving either ON but no myelitis or myelitis but no ON (1:1280 and 1:2560, respectively; Kruskal-Wallis *p* < 0.009; Dunn’s post test *p* < 0.05 for ON + myelitis vs. ON) (Fig. 5b).

### Impact of treatment status on MOG-IgG serum titers

Precise data on the treatment status at the time of blood sampling were available for 76/84 (90.5 %) MOG-IgG-positive samples. 28 samples were obtained during treatment with immunosuppressants (IS) or after PEX and 32 further samples were taken during or shortly after IVMP therapy (‘treated subgroup’); another 31 samples were taken prior to immunotherapy or in treatment-free intervals (‘untreated subgroup’). Treatments included IVMP, oral steroids, PEX, azathioprine, rituximab, methotrexate, mitoxantrone, natalizumab, and cyclosporine.

**Fig. 6:**
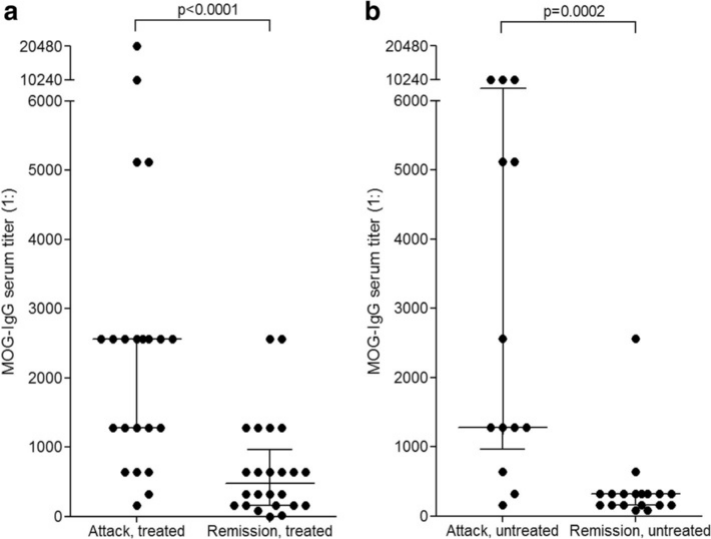
MOG-IgG titers and treatment status. While median MOG-IgG titers were lower during remission than during acute attacks in the treated subgroup (**a**), a similarly significant difference was also observed in the untreated subgroup (**b**). By contrast, no significant difference in median titers was observed between treated and untreated patients, neither during acute attacks nor during remission (not shown)

Median MOG-IgG serum titers differed significantly between relapse (1:2560, range 160–20480; *N* = 23) and remission (1:480, range 0–2560; *N* = 23) in patients treated with IS and/or PEX (*p* < 0.0001, Fig. 6a). However, a similar difference was present also in the untreated subgroup (*p* = 0.0002; Fig. 6b), suggesting that the decline in titers in the treated subgroup may have not been due only to treatment effects but may also reflect the natural disease course. In line with that notion, the median MOG-IgG titer in the treated subgroup did not differ significantly from that in the untreated subgroup, irrespective of whether all samples, only samples taken during relapse, or only samples obtained during remission are taken into account (data not shown).

Of note, 49/52 (94.2 %) samples were positive despite treatment with IS/PEX and/or steroids (or 25/28 [89.3 %] if only IS/PEX is considered). Of note, low MOG-IgG remained detectable in all four patients treated with rituximab at the time of blood sampling (titers 1:160–1:640). In a further patient treated with rituximab, a MOG-IgG titer of 1:1280 was documented during a relapse and was associated with recurrence of B cells.

### Frequency of CSF MOG-IgG

In total, 17 CSF samples from 15 MOG-IgG-seropositive patients from group I were available for testing. All but one sample were taken during an acute attack (median time since attack onset 10 days). Ten of those 17 CSF samples were obtained within 30 days after disease onset and 7 - including two follow-up samples - later in the disease course (range 71–5406 days after onset).

Twelve out of 17 CSF samples (71 %) were positive for MOG-IgG. The median CSF MOG-IgG titer was 1:4 (range 2–64). Individual CSF and serum results are shown in Table 3. Median titers did no differ between CSF samples taken at the time of the first attack (1:3) and CSF samples taken during an acute attack later in the disease course (1:3). Twelve out of 15 (80 %) patients were positive for CSF MOG-IgG at least once. In two of the three CSF-negative patients, lumbar puncture (LP) was delayed (1.5, 2 and 3 weeks, respectively, after attack onset) and was performed after or during IVMP therapy, respectively; and in all three, LP was done for acute isolated ON, a manifestation that was also associated with lower serum titers (Fig. 5a). Among the CSF positives, median CSF MOG-IgG titers in the initial sample taken during an acute attack were slightly higher in patients with acute myelitis (with or without concomitant ON and/or brainstem encephalitis) than in patients with acute ON (1:4 [range 2–64] vs. 1:1 [range 0–4]). An additional CSF sample obtained from the only serum MOG-IgG-positive control patient was negative for MOG-IgG.

In addition, 17 CSF samples from 17 control patients with RRMS were tested. All of those were negative for CSF MOG-IgG.

### CSF MOG-IgG in the long-term course

Follow-up CSF samples were available from two patients. In both cases, MOG-IgG were detectable in the CSF a few days after disease onset, at titers of 1:64 and 1:4, respectively, but not at repeat LP 51 and 21 days, respectively, later. One patient had been treated with IVMP, oral steroids, ten plasma exchanges, and IVIG in the meantime, the other one with IVMP alone. The decline in CSF titers was paralleled by a drop in serum titers from 1:10240 to 1:1280 and from 1:2560 to 1:1280, respectively, in these two patients.

### Origin of CSF MOG-IgG

**Fig. 7:**
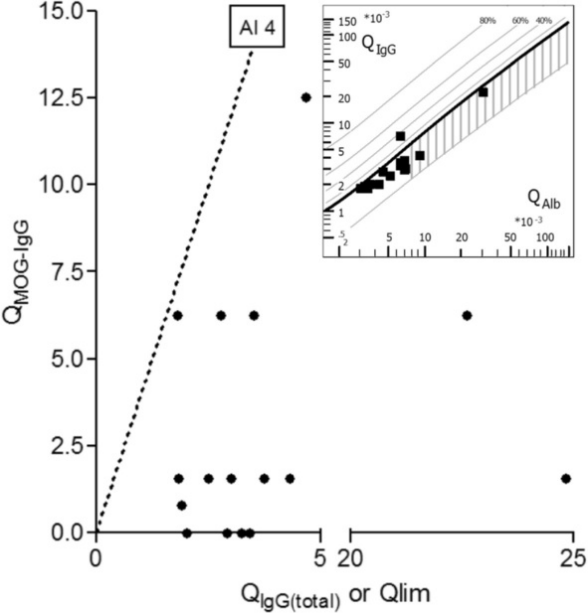
MOG-specific antibody index (AI). Calculation of the MOG-specific AI in 17 paired CSF/serum samples from 15 MOG-IgG-positive patients did not reveal evidence for intrathecal synthesis of MOG-IgG. The *dotted line* indicates the upper limit of the reference range (AI = 4). *Inset*: Reiber diagram [40] demonstrating absence of total IgG intrathecal synthesis in 16 samples from 14 patients and presence of blood-CSF barrier dysfunction in 6/17 samples. Q_IgG_ = CSF/serum total IgG ratio; Q_MOG-IgG_ = CSF/serum MOG-IgG ratio; Q_Alb_ = CSF/serum albumin ratio; Q_lim_ = upper reference range of Q_IgG_ (see methods section for details)

Seventeen paired CSF and serum samples were titrated to calculate the MOG-specific AI. Evidence for intrathecal IgG synthesis was present in none of these 17 samples: in 5 samples no MOG-specific IgG was detectable in the CSF, and in the remaining 12 samples the MOG-specific AI was <4 (Table 2, Fig. 7), indicating that MOG-IgG are produced mainly in the periphery and reach the CSF by passive diffusion or through a leaky blood-brain and/or blood-CSF barrier. In line with that finding, CSF-restricted total IgG oligoclonal bands were absent in 16/17 samples tested and Q_IgG(total)_ was below Q_lim_ in 16/17 cases, while Q_Alb_ exceeded the age-specific reference range in 6/17 (35.3 %) samples, indicating disruption of the blood-CSF barrier function.

**Table 2:** Lack of evidence for intrathecal IgG synthesis in 17 CSF samples from 15

Sample no.	MOG-IgG titer, serum	MOG-IgG titer, CSF	MOG-IgG titer required for AI >4	Evidence for intrathecal MOG-IgG synthesis
#1	1:10240	1:64	1:925.7	No
#2	1:2560	1:4	1:25.6	No
#3	1:320	1:2	1:2.3	No
#4	1:10240	1:16	1:152.5	No
#5	1:640	1:4	1:9	No
#6	1:2560	1:4	1:30.7	No
#7	1:10240	1:16	1:176.1	No
#8	1:2560	1:2	1:19.5	No
#9	1:320	1:2	1:3.6	No
#10	1:1280	1:2	1:9.4	No
#11	1:2560	1:4	1:25	No
#12	1:320	1:4	1:6	No
#13^a^	1:1280	NEG	1:17.4	No
#14	1:320	NEG	1:3.7	No
#15	1:1280	NEG	1:10.2	No
#16	1:160	NEG	1:2.1	No
#17^b^	1:1280	NEG	1:10.2	No

MOG-IgG seropositive patients with ON and/or myelitis. *NEG* negative. ^a^Follow-up to sample #1; ^b^ follow-up to sample #2

### MOG immunoglobulin class and subclass analyses

**Fig. 8:**
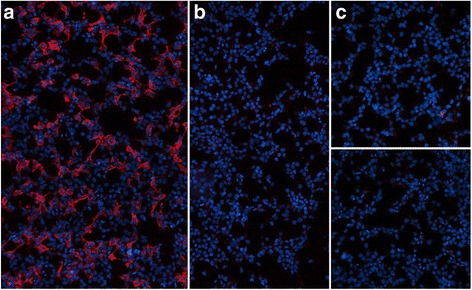
MOG-IgG1 as detected in the fixed-cell CBA. **a**, **b** Binding of serum IgG1 antibodies (from a patient with recurrent optic neuritis) to HEK293 cells transfected with human full-length MOG (**a**), but not to mock-transfected HEK293 cells (**b**). **c** Negative control serum (from a patient with RRMS) binding neither to the MOG-transfected cells (*upper panel*) nor to the mock-transfected control cells (*lower panel*). Bound patient IgG1 was detected by successive incubation with an unlabeled sheep anti-human IgG1 secondary antibody and an AlexaFluore®568-labeled donkey anti-sheep IgG antibody (*red fluorescence*). Cell nuclei were stained with 4’,6-diamidino-2-phenylindole (*blue fluorescence*)

Twenty serum samples, including 14 MOG-IgG-positive sera from 13 patients from group I (8 × relapse, 6 × remission) and 6 control sera from group III patients, were tested for MOG-IgG1 using the fixed-cell CBA (Euroimmun). All 14 group I samples were positive for MOG-IgG1; by contrast, none of 6 control sera contained MOG-IgG1 antibodies (Fig. 8). MOG-IgG1 was also present in the CSF in 3/3 MOG-IgG serum positive patients tested.

In addition, 20 MOG-IgG-positive samples from 15 patients of group I were tested for MOG-IgM and MOG-IgA using the fixed-cell assay. Of these, only 2 samples (from a patient with a history of ON and myelitis) were positive for MOG-IgM and none for MOG-IgA (Table 3).

**Table 3:** MOG-IgG, MOG-IgG1, MOG-IgM, and MOG-IgA results from 21 samples

No	MOG-IgG1	MOG-IgG	MOG-IgM	MOG-IgA	Disease status
1	POS	POS	NEG	NEG	Relapse
2	POS	POS	NEG	NEG	Relapse
3A	POS	POS	NEG	NEG	Relapse
3B	POS	POS	NEG	NEG	Remission
4	POS	POS	NEG	NEG	Remission
5	POS	POS	NEG	NEG	Remission
6	POS	POS	NEG	NEG	Remission
7	POS	POS	NEG	NEG	Remission
8	POS	POS	NEG	NEG	Relapse
9A	POS	POS	NEG	NEG	Relapse
9B	n.d.	POS	NEG	NEG	Remission
10A	POS	POS	NEG	NEG	Relapse
10B	n.d.	POS	NEG	NEG	Remission
11	n.d.	POS	NEG	NEG	Relapse
12	n.d.	POS	NEG	NEG	Remission
13	n.d.	POS	NEG	NEG	Remission
14A	n.d.	POS 1:1000	POS 1:20	NEG	Relapse
14B	n.d.	POS 1:100	POS 1:10	NEG	Remission
15A	n.d.	POS	NEG	NEG	Remission
15B	n.d.	POS	NEG	NEG	Remission
16	POS	POS	n.d.	n.d.	Relapse
17	POS	POS	n.d.	n.d.	Relapse
18	POS	POS	n.d.	n.d.	Relapse

MOG-IgG was determined using a commercial fixed CBA (cut-off 1:10). MOG-IgG1 was also present in 3/3 CSF samples from MOG-IgG1-seropositive patients (not shown). *POS* positive, *NEG* negative, *n.d* not done

## Discussion

In 2011, some of us reported for the first time on serum autoantibodies to full-length human MOG in patients with NMO and related disorders [17]. This finding was later independently confirmed by several groups [18–25, 27, 46]. However, some previous analyses were hampered by low patient numbers and short follow-up times, lack of CSF samples, and, in some cases, uncertainty regarding assay specificity due to low control sample numbers. Moreover, some studies included no Caucasian patients. Here, we report on serological findings from a large cohort of MOG-IgG-positive patients, almost all of Caucasian origin. Our study demonstrates (i) that MOG-IgG are associated with ON and myelitis in a substantial proportion of cases; (ii) that MOG-IgG and AQP4-IgG do not usually co-exist in patients with ON and/or myelitis, which is in support of the notion of MOG-IgG being denoting an entity distinct from AQP4-IgG-positive NMO spectrum disorder (NMOSD) [47]; (iii) that MOG-IgG are present already at the very onset of disease, which argues against MOG-IgG being a secondary epiphenomenon; (iv) that MOG-IgG remain detectable in the long-term course of the disease, indicating that the antibodies, if pathogenic, may not only trigger the disease but remain relevant in the long run; persisting MOG-IgG antibodies have also been described in pediatric patients diagnosed with relapsing demyelinating disease [26, 28]; (v) that MOG-IgG persist also during remission in the majority of patients, which is similar to what has been reported in AQP4-IgG-positive NMOSD [48], is important from a diagnostic point of view, and suggests that MOG-IgG alone is not sufficient to induce disease activity but other factors, such as an increase in titers, impaired blood-CSF barrier function (elevated Q_Alb_ was indeed noted in 12/36 (32.4 %) patients in the total cohort [36]) or T-cells, may be required; (vi) that, similar to AQP4-IgG [48], MOG-IgG serum titers depend on disease activity, with significantly higher median titers during acute attacks than during remission, both in treated and in untreated patients, further supporting a potential pathogenic role of MOG-IgG; (vii) that absolute serum MOG-IgG titers vary substantially inter- and intraindividually, both during acute disease and during remission, with no clear cut-off for relapse induction; (viii) that MOG-IgG serum titers may also vary significantly with clinical presentation and, in some cases, treatment; (ix) that MOG-IgG (similar to AQP4-IgG [48]) may remain detectable even during treatment with rituximab, which suggests a role of long-lived plasma cells not affected by CD20-targeted immune therapy in the production of MOG-IgG and, given that no attacks occurred in the four patients tested in this study while on active treatment with that drug, that persistence of low-titer MOG-IgG does not per se argue against the efficacy of rituximab; (x) that MOG-IgG (like AQP4-IgG [42]) is detectable in the CSF in a substantial number of patients during acute attacks; this is in line with a small previous study by Dale et al., who found MOG-IgG in 2/4 patients positive for serum MOG-IgG [49]; (xi) that CSF MOG-IgG (just like CSF AQP4-IgG and in line with the lack of CSF-restricted oligoclonal bands (OCB) in most MOG-IgG-positive patients as shown in part 2 [36]) is mainly of extrathecal origin, i.e., enters the CNS from the systemic circulation, which may be of therapeutic relevance [42, 50–52]; (xii) that both serum and CSF MOG-IgG belong to the complement-activating IgG1 subclass (just as AQP4-IgG does [53, 54] and in agreement with the presence of complement deposits in CNS lesions in MOG-IgG-positive patients [31, 32]), again supporting the notion of MOG-IgG being of pathogenetic relevance; and, last but not least, (xiii) high specificity of the live CBA used in the present study [17] based on a very large series of control samples, which is important since it affirms the validity of results obtained in previous studies that have employed that assay [25, 26, 55, 56].

Our study features strengths and limitations. Among the strengths of the study we count (a) the high number of MOG-IgG-positive patients with ON and/or myelitis identified and analyzed (*N* = 50) compared with previous studies (median 9 patients in [17–25, 27, 46]); (b) the availability of a relevant number of follow-up or stored serum samples; (c) the availability of both samples taken at the very onset of the disease and samples taken more than a decade thereafter; (d) the availability of a substantial number (*N* = 17) of paired CSF and serum samples; (e) the inclusion of a relevant number of MOG-IgG-positive samples from untreated patients (*N* = 31); the fact (f) that virtually all patients were of Caucasian origin; (g) that the study was performed using a multicenter (*N* = 11) approach, thereby reducing potential center-specific selection biases; (h) that all MOG-IgG-positive patients were seen at university centers with specialized neuroimmunology departments, thereby potentially increasing diagnostic accuracy; (i) that detailed data on disease activity, clinical presentation, and treatment status at the time of blood sampling were available for most patients; (j) that both MOG-IgG and AQP4-IgG results were available from a relevant number of patients (*N* = 459); (k) that an already well-established CBA with published sensitivity and specificity [17] was used for MOG-IgG testing; (l) that a very large number of controls, interspersed in a random pattern, were included to re-validate the specificity of that assay (*N* = 222); (m) that all low-titer samples (1:160, 1:320) were confirmed using a second, methodologically independent CBA; and (n) that samples were evaluated by investigators not involved in patient recruitment and blinded to all clinical data.

The limitations include a potential referral bias due to the possibility that patients with ON and/or myelitis may have been preferentially referred for MOG-IgG testing as a consequence of the close association of MOG-IgG with these two conditions reported in the previous literature [18–28, 57]. However, MOG-IgG has also been reported in, mostly pediatric, patients with acute disseminated encephalomyelitis (ADEM). Although ADEM was considered as a differential diagnosis by the initially treating physicians in a few patients in our series (see part 2 [36] for details), our study did not specifically focus on children or on patients with a diagnosis of ADEM. Second, while the multicenter approach involving 11 specialized university departments is a potential strength as outlined above, it also carries the potential risk of a bias towards more severely affected patients. However, that risk is inherent to all tertiary care studies and cannot be completely avoided. It is important in this context that all centers involved in the present study also have specialized neuroinflammatory outpatient departments and that patients were recruited among both inpatients and outpatients. Finally, the threshold for admission is low in Germany, where public healthcare is free. In fact, a mild disease course was noted in a substantial proportion of patients (see part 2 [36] for details).

There is a discrepancy between the lack of MOG-IgG in the MS control group in this study and the fact that MS had been suspected by the then treating physicians at least once in 16/45 (35.6 %) MOG-IgG positive patients, as outlined in part 2 of this series [36]. This discrepancy may highlight differences in diagnostic accuracy between carefully defined study cohorts comprising patients diagnosed at specialized centers and everyday clinical practice at primary or secondary care level. This notion is supported by the fact that MS had been initially considered in 11 of those 16 patients despite a lack of CSF-restricted OCB, a diagnostic hallmark of MS (see part 2 [36] for details). Similarly, 10 MOG-IgG-positive patients who formally met the 2010 McDonald criteria for MS had no OCBs. Moreover, 11 patients with suspected MS had LETM lesions, which are usually absent in MS, and 11 did not meet Barkhof’s MRI criteria for MS. Finally, 6 patients in whom MS had been previously suspected did not meet the 2010 McDonald criteria (see part 2 [36]). With the discovery of AQP4-IgG [1, 58–60], MOG-IgG [17], *N*-methyl-D-aspartate receptor-IgG [61], and a plethora of often non-paraneoplastic autoantibodies identified in acute CNS inflammation over the past decade [62–66], including in patients with primary or secondary demyelination, it becomes increasingly clear that not all patients presenting with relapsing CNS disease of putative autoimmune etiology have classical MS–even if they formally meet the ‘positive’ clinicoradiological criteria for MS [67]. In fact, 50 % of the MOG-IgG-positive patients in this study had clinical or radiological involvement of the brain in addition to ON and/or myelitis and the Barkhof and McDonald criteria for multiple sclerosis (MS) were met by 15 % and 33 %, respectively, as shown in parts 2 and 3 of this series [36, 37]. MOG-IgG-positive patients, in whom the disease starts with isolated brain or brainstem involvement are particularly challenging [27, 36, 37, 68]. Thus more and more importance attaches to carefully considering the ‘negative’ criterion of ruling out other diagnoses (“no better explanation”) included in the current diagnostic consensus criteria for MS [69]. It also suggests that re-including CSF analysis in the diagnostic criteria for MS, as previously recommended by us and others [70], might help to improve diagnostic accuracy in patients with suspected MS.

It is of clinical relevance that 15/28 (53.6 %) of the MOG-IgG-positive patients with a history of myelitis identified in this study had recurrent attacks of myelitis [36]. If only patients with MOG-IgG-positive isolated myelitis are considered, 4/6 had recurrent myelitis and two had monophasic myelitis [36]. This suggests that MOG-IgG testing should be considered both in patients with monophasic and in patients with recurrent myelitis. Similarly, MOG-IgG was found both in patients with a single attack of ON and in patients with recurrent ON.

While treatment with IS was followed by a decline in relapse rate in individual patients, as outlined in part 2 of this series [36], no clear effect of IS on median MOG-IgG titers could be demonstrated in the present study. However, this is not totally surprising: while our study is among the largest in the field, patient numbers might still have been too low to detect such effects, especially when taking into account the large number of confounders such as disease activity, attack severity, clinical presentation, type and duration of treatment, drug-specific latency periods, and time since attack onset. Prospective studies with fixed sampling intervals and defined treatment regimens are highly warranted to assess the effect of immunotherapy on MOG-IgG titers and its impact on outcome and prognosis in a definite way.

## Conclusion

In summary, our study provides evidence supporting a potential pathogenic role of MOG-IgG, and thus the notion of MOG-IgG denoting a disease entity in its own right, by demonstrating in the largest cohort of patients so far: (i) a close association of MOG-IgG with a specific clinical phenotype (i.e., ON and/or myelitis); (ii) an increase in serum MOG-IgG titers during acute attacks; (iii) the presence of MOG-IgG in the CSF in the early phase of acute attacks in untreated patients; (iv) the presence of complement-activating anti-MOG antibodies of the IgG1 subclass both in the serum and in the CSF; and (v) absence of AQP4-IgG, an already well-established cause of optic nerve and spinal cord damage, in MOG-IgG-positive patients. Detailed clinical and paraclinical data were available for all 50 MOG-IgG-positive patients with ON and/or myelitis identified in this study and are comprehensively analyzed in parts 2 [36], 3 [37] and 4 [38] of this series.

## Abbreviations

ADEM: acute disseminated encephalomyelitis
AI: antibody index
AQP4: aquaporin-4
CBA: cell-based assay
CSF: cerebrospinal fluid
ELISA: enzyme-linked immunosorbent assay
IgG: immunoglobulin G
IM: immunomodulatory
IS: immunosuppressive
IVIG: intravenous immunoglobulins
IVMP: intravenous methylprednisolone
LETM: longitudinally extensive transverse myelitis
LP: lumbar puncture
MOG: myelin oligodendrocyte glycoprotein
MS: multiple sclerosis
NMO: neuromyelitis optica
NMOSD: neuromyelitis optica spectrum disorder
OCB: oligoclonal bands
OCT: optical coherence tomography
ON: optic neuritis
PEX: plasma exchange
PPMS: primary progressive MS
Q_Alb_: albumin CSF/serum quotient
Q_IgG_: IgG CSF/serum quotient
RRMS: relapsing remitting MS
SPMS: secondary progressive MS
